# *Connarus semidecandrus* Jack Exerts Anti-Alopecia Effects by Targeting 5α-Reductase Activity and an Intrinsic Apoptotic Pathway

**DOI:** 10.3390/molecules27134086

**Published:** 2022-06-24

**Authors:** Won Young Jang, Dong Seon Kim, Sang Hee Park, Ji Hye Yoon, Chae Yun Shin, Lei Huang, Ket Nang, Masphal Kry, Hye-Woo Byun, Byoung-Hee Lee, Sarah Lee, Jongsung Lee, Jae Youl Cho

**Affiliations:** 1 Department of Integrative Biotechnology, Sungkyunkwan University, Suwon 16419, Korea; wybest0327@naver.com (W.Y.J.); wetdry20@hanmail.net (D.S.K.); 2 Department of Biocosmetics, Sungkyunkwan University, Suwon 16419, Korea; 84701@naver.com (S.H.P.); kws05251@naver.com (J.H.Y.); shina8059@naver.com (C.Y.S.); ruidan0909@naver.com (L.H.); 3 Forestry Administration, Phnom Penh 120206, Cambodia; nang_keth@yahoo.com (K.N.); masphalsnu@gmail.com (M.K.); 4 National Institute of Biological Resources, Environmental Research Complex, Incheon 22689, Korea; hwbyun@korea.kr (H.-W.B.); dpt510@korea.kr (B.-H.L.)

**Keywords:** *Connarus semidecandrus* Jack, androgenic alopecia, anti-alopecia, androgen receptor, 5-α reductase, programmed cell-death, Bcl-2

## Abstract

There is a growing demand for hair loss treatments with minimal side effects and recurrence potential. *Connarus semidecandrus* Jack has been used as a folk medicine for fever in tropical regions, but its anti-alopecia effects remain unclear. In this study, the anti-androgenic alopecia effect of an ethanol extract of *Connarus semidecandrus* Jack (Cs-EE) was demonstrated in a testosterone-induced androgenic alopecia (AGA) model, in terms of the hair–skin ratio, hair type frequency, and hair thickness. The area of restored hair growth and thickened hair population after Cs-EE treatment showed the hair-growth-promoting effect of Cs-EE. Histological data support the possibility that Cs-EE could reduce hair loss and upregulate hair proliferation in mouse skin by shifting hair follicles from the catagen phase to the anagen phase. Western blotting indicated that Cs-EE reduced the expression of the androgenic receptor. Cs-EE treatment also inhibited programmed cell death by upregulating Bcl-2 expression at the mRNA and protein levels. The anti-alopecia effect of Cs-EE was confirmed by in vitro experiments showing that Cs-EE had suppressive effects on 5-α reductase activity and lymph node carcinoma of the prostate proliferation, and a proliferative effect on human hair-follicle dermal papilla (HDP) cells. Apoptotic pathways in HDP cells were downregulated by Cs-EE treatment. Thus, Cs-EE could be a potential treatment for AGA.

## 1. Introduction

Alopecia is the most common disease known to cause hair loss or hair thinning irrespective of sex [1,2], in addition to being a side effect of chemotherapy [3]. The disease is not life threatening, but it commonly leads to depression, which lowers quality of life [4,5]. The mechanism of androgenic alopecia (AGA) in particular has not been fully identified, but hair growth inhibition due to the androgenic receptor (AR) signaling system is believed to be the biggest cause [6].

The androgens are endogenous steroid hormones: testosterone, androstenedione, dehydroepiandrosterone, and dihydrotestosterone (DHT). AR can bind testosterone and DHT, but DHT has a five-fold higher affinity for AR than testosterone does [7]. AR activated by DHT is known to regulate downstream genes that cause hair loss progression [8,9,10]. In fact, flutamide, which acts as an AR antagonist, and finasteride, which suppresses 5-α reductase (that regulates the conversion of testosterone to DHT) are already used to suppress AGA [11,12]. However, the possibility of recurrence, the lengthy duration of treatment required for effectiveness, and side effects such as diabetes, insulin resistance, and the deterioration of sexual function caused by changing the systemic steroid metabolism, mean that better treatments for AGA are needed [13,14,15,16].

Hair follicles continuously undergo four-phase cycles: anagen (growth), catagen (involution), telogen (rest), and exogen (shedding). Catagen occurs with apoptosis of the hair matrix, dermal papilla cells, inner root sheath keratinocyte, and outer root sheath keratinocytes [17]. The canonical pathway of apoptosis is controlled by the anti-apoptotic Bcl-2 family (Bcl-2, Bcl-XL, and Bcl-W) and the pro-apoptotic Bcl-2 family (Bax, Bak, and Bad) [18]. During catagen, the Bcl-2/Bax ratio decreases dramatically compared with anagen levels. That shift triggers the release of cytochrome c and induces procaspase 9, an apoptosis initiator enzyme, to become caspase 9, which promotes the activity of caspase 3, which in turn induces apoptosis by upregulating the proteolysis of the cytoplasmic substrate and DNA fragmentation, as mediated by DNase [19]. Because one of the representative causes of hair loss is the failure to control apoptosis during catagen, resulting in the continuous induction of apoptosis regression, a material with anti-apoptotic properties could be developed as a potential treatment for AGA [20].

*Connarus semidecandrus* Jack, a species of the *Connaraceae* family, is a flowering plant spread widely throughout the tropics, including Cambodia, Laos, and Malaysia. The therapeutic potential of species from the *Connaraceae* family has been previously reported [21]. *C. semidecandrus* has been used as a traditional medicine for fever relief [22]. The antipyretic activity of *C. semidecandrus* was confirmed using yeast-induced fever models in rats [23]. No previous research has considered the effect of *C. semidecandrus* on hair loss. Therefore, we investigated the novel anti-alopecia effects of an ethanol extract of the whole *C. semidecandrus* plant (Cs-EE) in human lymph node carcinoma of the prostate (LNCaP) cells, human hair follicle dermal papilla (HDP) cells, and in vivo murine models of testosterone-induced AGA.

## 2. Results

### 2.1. Phytochemical Components of C. semidecandrus

**Figure 1:**
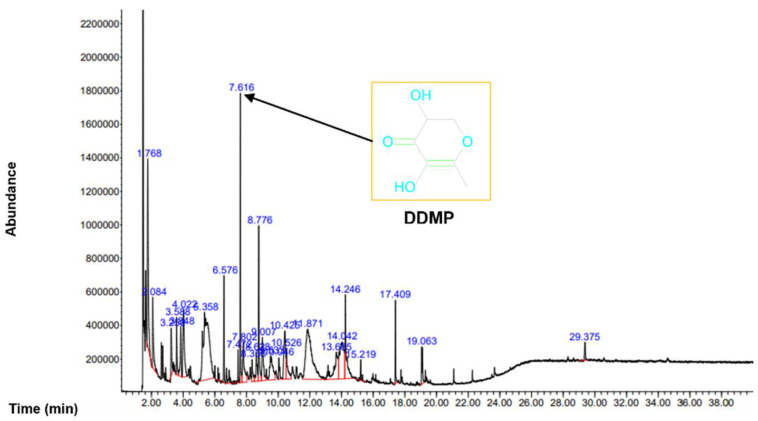
GC-MS was conducted to analyze the phytochemicals in Cs-EE.

To analyze the organic compounds in *C. semidecandrus*, gas chromatography–mass spectrometry (GC-MS) was conducted (Figure 1).

The most abundant compound was 4H-pyran-4-one,2,3-dihydro-3,5-dihydroxy-6-methyl (DDMP). Because the leaves of Punica granatum L., which are known to have hair promoting activity, share components such as maltol and DDMP with *C. semidecandrus* [24], we examined the regulatory effects of *C. semidecandrus* on hair growth. All the compounds in Cs-EE are listed in Table 1.

### 2.2. Hair Growth Promoting and Hair Thickening Effects of Cs-EE

**Figure 2:**
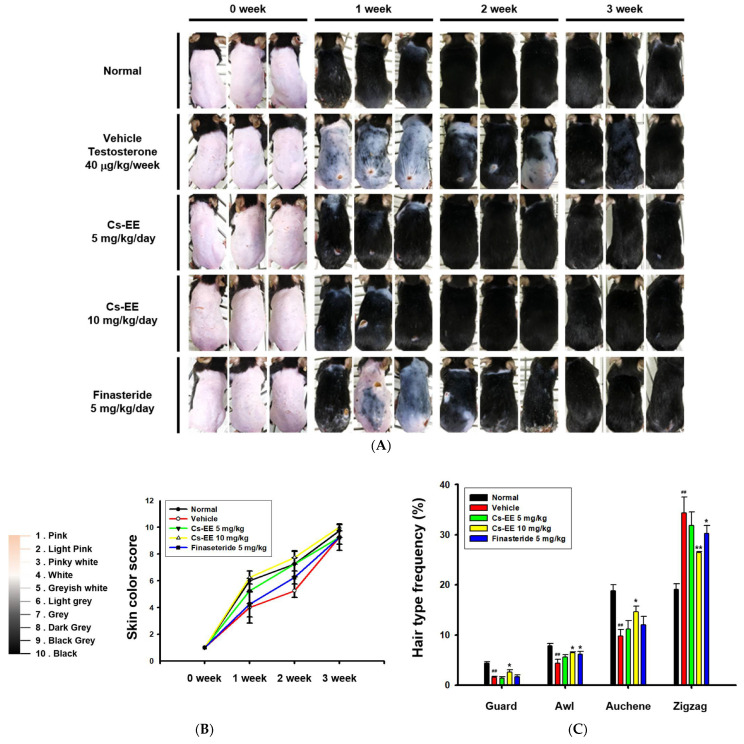

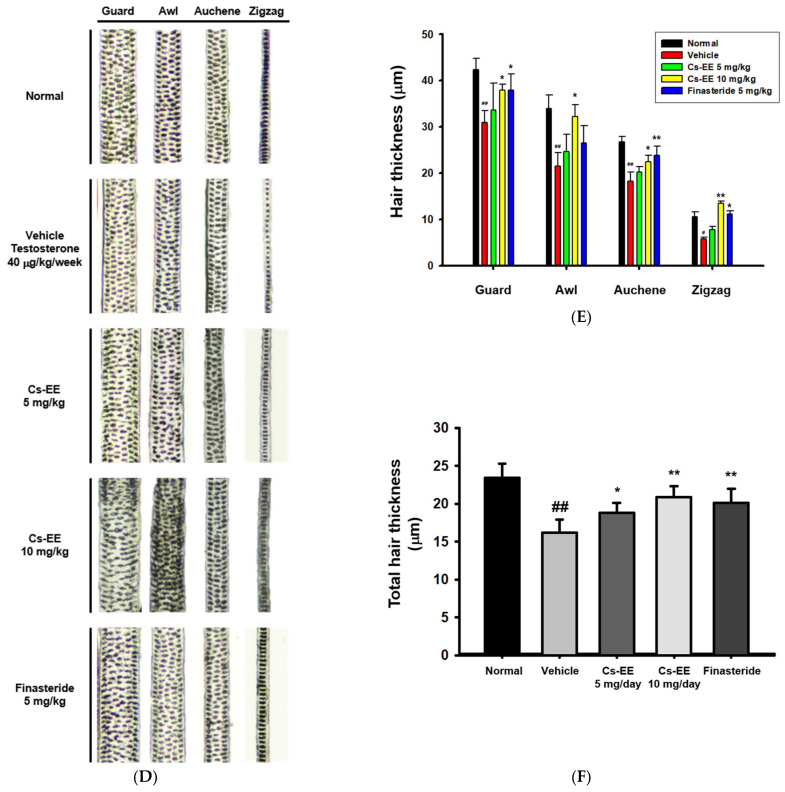
Effects of Cs-EE on the testosterone-induced androgenic alopecia model. (**A**) Morphological observations of mouse backs were collected for three weeks, and (**B**) skin color scores were assigned. (**C**) Hair type frequency (guard, awl, auchene, and zigzag) was measured in each group. (**D**) Photos of each hair type were collected from the five groups for each hair thickness. (**E**,**F**) The total hair thickness of each group was measured. ^#^: *p* < 0.05 and ^##^: *p* < 0.01 compared with the normal group, * *p* < 0.05 and ** *p* < 0.01 compared with the vehicle group (treated with testosterone).

For analysis of the anti-alopecia effect of Cs-EE, a testosterone-induced AGA mouse model was developed. Three weeks after being shaved, the mice treated with Cs-EE showed remarkable regrowth, with almost complete recovery of skin color. In fact, the speed of the recovery was faster than in the vehicle and finasteride groups. Most of the shaved skin remained hairless in the vehicle and finasteride groups after week 1, whereas the Cs-EE group showed clear hair growth (Figure 2A).

As shown in the photos collected, color from pink to black was used to determine skin color scores (Figure 2B). The skin color scores of the Cs-EE group were evidently higher than those in the vehicle and finasteride groups at every time point, and were in fact similar to the normal group. Thus, Cs-EE reliably promoted hair regrowth to the extent that the Cs-EE group was similar to the normal group. The hair type frequency also changed significantly, with the normal group showing an increase in the number of thick guard type hairs, and the testosterone group showing an increase in the number of thin zigzag type hairs, which was alleviated by Cs-EE (Figure 2C). Hair thickness was measured using photos of each hair type (Figure 2D), which confirmed that the thickness of all types of hair in the vehicle group was relatively thin, and the thickness of each hair type was increased by Cs-EE treatment (Figure 2E). The total hair thickness, which was decreased by a subcutaneous injection of testosterone, was also recovered by Cs-EE treatment (Figure 2F).

### 2.3. Histological Analysis and Ex Vivo Culture of Mouse Hair Follicles Reveal Hair Protective and Growth Effects of Cs-EE

**Figure 3:**
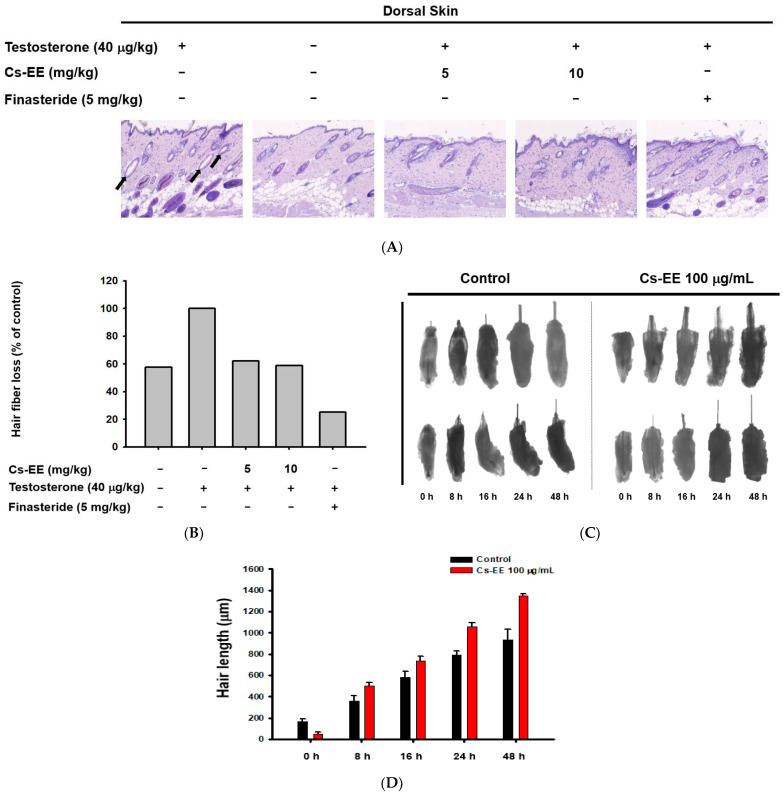
Effect of CS-EE on hair growth within ex vivo mouse hair follicles. (**A**) H&E staining of mouse skin tissue under 4× magnification. (**B**) The ratio of hair fibers that degraded (black arrows in (**A**)) to all hair fibers was calculated and expressed. (**C**) Representative images of mouse hair follicles cultured in dermal papilla cell medium with DMSO vehicle or Cs-EE (100 μg/mL). (**D**) The length of hair in each follicle was recorded and compared.

The histological data suggest that the hair follicles of the testosterone group were empty, with no hair fibers (black arrows in Figure 3A).

However, Cs-EE treatment triggered the development of new hair fibers on the epidermal surface (Figure 3A,B). Ex vivo experiments on mouse hair follicles confirmed the hair-growth-promoting effect of Cs-EE: a 48-h incubation of hair follicles with Cs-EE (100 μg/mL) increased the hair length to more than 1200 μm, whereas the hair length reached less than 1000 μm in the absence of Cs-EE (Figure 3C,D). These results indicate that Cs-EE inhibits testosterone-mediated hair loss and induces hair growth.

### 2.4. Cs-EE Reduces AR Level and Inhibits Apoptotic Pathway In Vivo

**Figure 4:**
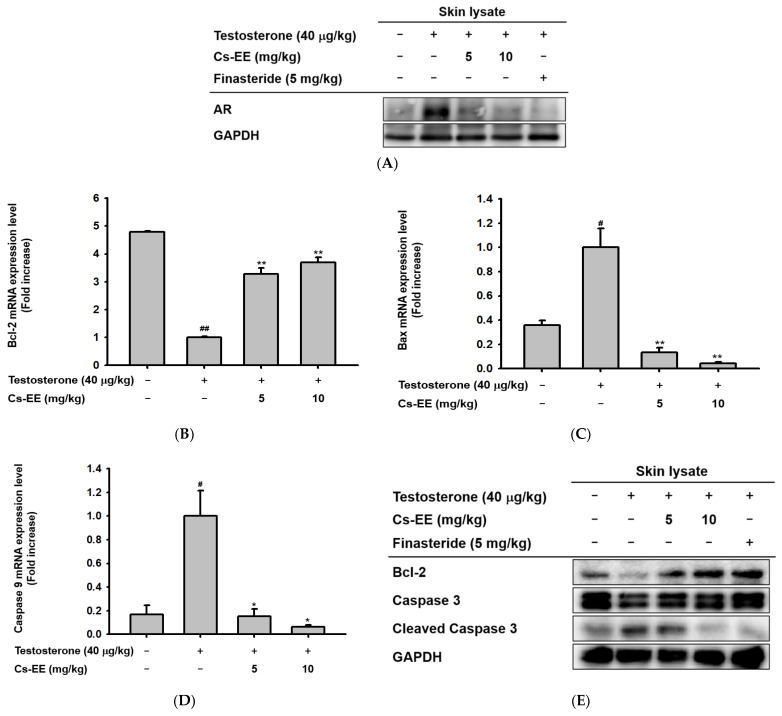
Androgen and apoptosis inhibition efficacy of Cs-EE at both the mRNA and protein levels in a testosterone-induced alopecia mouse model. (**A**) The protein level of androgenic receptor was also confirmed from skin lysate. GAPDH was used as a control protein. (**B**–**D**) The mRNA expression levels of Bcl-2, Bax, and caspase 9 in skin lysate were measured using quantitative real-time PCR. GAPDH was used as a control gene. € The protein level of Bcl-2 and the activation of caspase 3 in skin lysate were also determined by Western blotting. All data (**B**–**D**) are expressed as the mean ± standard deviation. ^#^ *p* < 0.05 and ^##^: *p* < 0.01 compared with the normal group, * *p* < 0.05 and ** *p* < 0.01 compared with the Cs-EE-untreated group.

To find the mechanism of the anti-alopecia effect of Cs-EE, the protein levels of AR in the skin on the backs of the mice were detected by Western blotting. The Western blotting analysis (Figure 4A) showed that Cs-EE had an inhibitory effect on AR expression, suggesting that it has an anti-androgenic effect.

Because one of the important factors of hair loss is early termination of hair follicle growth due to apoptosis, [25]; we also checked the mRNA expression of Bcl-2, Bax, and caspase 9 in the mouse skin tissue. Interestingly, the mRNA expression level of Bcl-2 was increased after Cs-EE treatment (Figure 4B). On the other hand, the mRNA expression of genes with pro-apoptotic properties, including Bax and caspase 9, was reduced considerably after Cs-EE treatment under testosterone conditions (Figure 4C,D). The Western blotting analysis showed results consistent with the real-time PCR results: a lower protein level of cleaved caspase 3 was detected in the Cs-EE group after testosterone induction, and the Bcl-2 protein level recovered after Cs-EE application (Figure 4E). These results show that the protective effect of Cs-EE on hair derives from its downregulation of AR and apoptotic pathways.

### 2.5. Cs-EE Exhibits Opposite Effects on the Survival and Proliferation of LNCaP and HDP Cells

**Figure 5:**
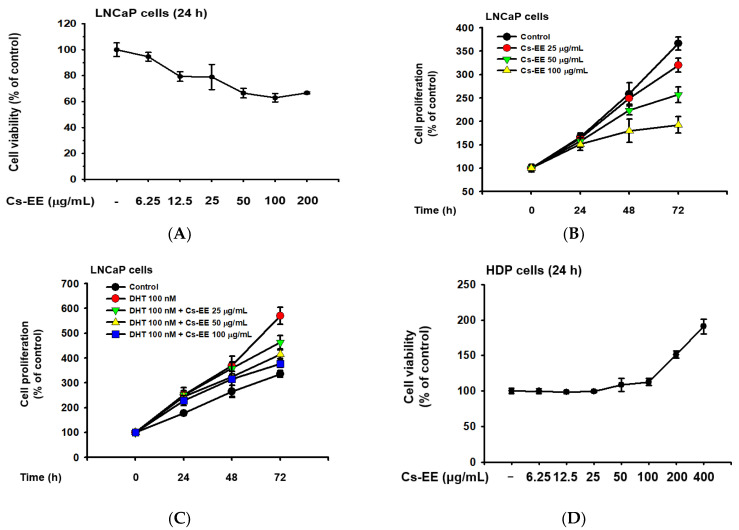

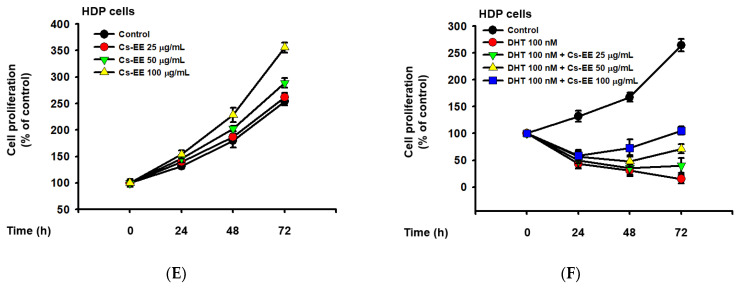
Cs-EE inhibited the survival and proliferation of an androgen-dependent prostate cancer cell line (LNCaP) and upregulated the survival and proliferation of human dermal papilla (HDP) cells. (**A**) Cell viability assay using MTT solution with different Cs-EE concentrations. (**B**,**C**) LNCaP proliferation was detected in both the DHT-treated and untreated conditions in 0, 25, 50, and 100 μg/mL of Cs-EE. (**D**) The cytotoxicity of Cs-EE on HDP cells was measured using the MTT assay. (**E**,**F**) HDP proliferation in different concentrations of Cs-EE was evaluated in the DHT-treated and -untreated conditions.

LNCaP is an androgen-sensitive human prostate cancer cell line, and the upregulation of LNCaP proliferation after DHT treatment has previously been reported [26,27]; therefore, we conducted cell viability and cell proliferation assays to evaluate the anti-androgenic effect of Cs-EE. Up to a concentration of 12.5 μg/mL, Cs-EE showed toxicity against LNCaP cells, lowering their viability to less than 80% (Figure 5A).

Cs-EE at 100 μg/mL reduced LNCaP proliferation from more than 350% of the control to less than 200% (Figure 5B). Surprisingly, the upregulation of LNCaP proliferation caused by DHT (100 nM) treatment was reduced by Cs-EE treatment in a time- and dose-dependent manner (Figure 5C).

We also measured the cytotoxic effect of Cs-EE on HDP cells. Interestingly, up to 100 μg/mL of Cs-EE had no significant effect in cell viability, and treatment of more than 100 μg/mL of Cs-EE drastically increased cell viability (Figure 5D). Compared to the absence of Cs-EE, HDP proliferation also increased (Figure 5E). Moreover, 100 nM DHT had cytotoxic effects on HDP cells, and Cs-EE co-treatment recovered the proliferation of HDP cells up to 100% (Figure 5F). These results indicate that Cs-EE acted as an anti-alopecia agent by blocking the androgenic pathway and protecting HDP cells from death.

### 2.6. Cs-EE Promotes 5α-Reductase Inhibition and Anti-Apoptotic Processes in HDP Cells

**Figure 6:**
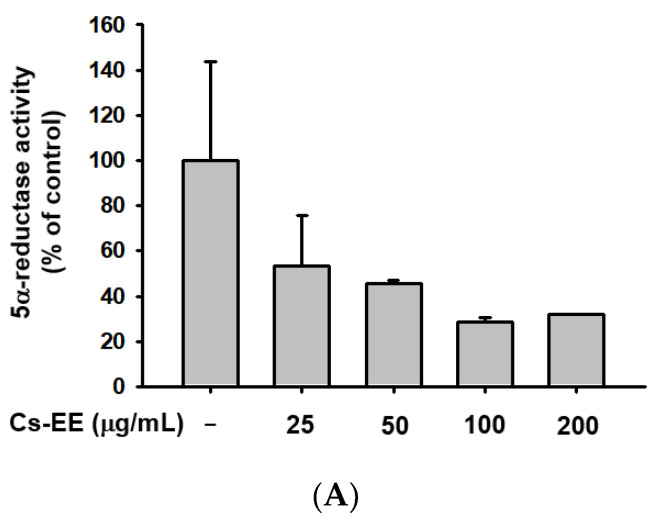

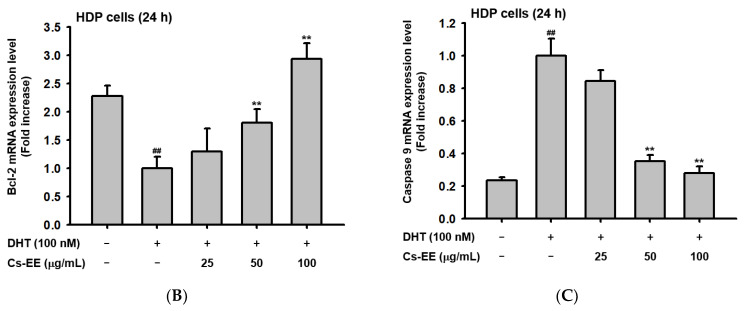
Cs-EE downregulated 5α-reductase activity and regulated the mRNA expression of apoptotic genes in HDP cells. (**A**) The activity of 5α-reductase in Sprague Dawley rats was checked with several concentrations of Cs-EE. (**B**,**C**) Quantitative real-time PCR was performed to assess the gene regulation effect of Cs-EE on apoptotic pathways. ^##^ *p* < 0.01 compared with the normal group ** *p* < 0.01 compared with the DHT-treated group.

We conducted a 5α-reductase activity assay to find the mechanism of the anti-androgenic activity of Cs-EE (Figure 6A). 

More than 100 μg/mL of Cs-EE reduced 5α-reductase activity to less than 40% of normal levels. Therefore, the Cs-EE-mediated AR protein level reduction and the cytotoxicity against LNCaP cells were driven by the inhibitory effect that Cs-EE has on 5α-reductase activity.

To confirm the anti-alopecia effect of Cs-EE in vitro, HDP cells were treated with DHT (100 nM) after Cs-EE pre-treatment. The reduced mRNA level of Bcl-2 was dose-dependently increased by Cs-EE treatment (Figure 6B). On the other hand, the mRNA level of caspase 9, which is a pro-apoptotic agent, was decreased by Cs-EE (Figure 6C). The real-time PCR data reaffirmed that the anti-hair-loss effect of Cs-EE occurred through the regulation of the cell-death signaling pathway.

## 3. Discussion

*Connarus semidecandrus* is widely used in tropical regions to treat fever or muscle pain [23,28]. However, no previous study has evaluated its therapeutic potential in other disease models. GC-MS analysis revealed that *C. semidecandrus* contains maltol and DDMP, indicating that it might have hair-growth-promoting effects [24]. Therefore, we evaluated the protective effects of an ethanol extract of *C. semidecandrus* against hair loss.

Alopecia induced by testosterone in an animal model was relieved by Cs-EE; hair recovery speed and hair thickness both increased after Cs-EE treatment. Histological data also demonstrated that Cs-EE had a protective effect on hair follicles. The results from an ex vivo experiment using mouse hair follicles were consistent with the data from the alopecia model showing that Cs-EE has a hair-growth-promoting effect.

Androgens, including testosterone, androstenedione and DHT, trigger hair loss, which is known as AGA [29]. DHT has the highest affinity for AR among the androgens, and AR activated by its interaction with DHT can translocate to the nucleus for further gene expression [30]. Therefore, we measured the protein expression of AR to confirm the potential utility of Cs-EE. That result showed a significant reduction in AR protein levels after Cs-EE treatment. Moreover, Cs-EE blocked the survival and DHT-dependent proliferation of LNCaP cells. Therefore, we assumed that Cs-EE regulates reactions upstream of AR activation. The one-step conversion of testosterone to the highly attractive DHT requires the help of 5α-reductase [31]. Interestingly, Cs-EE significantly interfered with 5α-reductase activity at every tested concentration, operating as an androgen-mediated signaling pathway regulator.

Failure to shift from the catagen/telogen phase to the anagen phase causes continuous apoptotic signaling that shrinks and degrades hair follicles, eventually resulting in insufficient hair [32]. The most well-known apoptotic pathway is the intrinsic pathway, which is mainly mediated by mitochondria [33]. That process begins with the translocation of Bax to the mitochondrial membrane, which triggers the release of cytochrome c and stops Bcl-2, which otherwise blocks the entrance of cytochrome c to the cytosol. The released cytochrome c binds to procaspase 9 and other complexes to form an apoptosome. The apoptosome then activates the caspase 3 signaling cascade, which induces cell demolition [34,35,36]. We examined the anti-apoptotic effect of Cs-EE in vivo and in vitro. After Cs-EE treatment, the mRNA levels of Bcl-2, which blocks apoptosis, increased, and the mRNA levels of Bax and caspase 9, which activate apoptosis, decreased. Cs-EE also increased the protein level of Bcl-2 and suppressed the protein level of cleaved caspase 3 in vivo, which indicates that Cs-EE treatment halted the caspase signaling cascade. That discovery suggests that Cs-EE might be useful as a drug to ameliorate unbalanced apoptotic responses.

Cs-EE could have clinical efficiency against alopecia by regulating either the androgenic axis or the apoptotic axis, which emphasizes its therapeutic potential. Clinical trials to inhibit the cell-death pathway using Bcl-2 antisense oligonucleotides, caspase inhibitors and synthetic p53 have been conducted continuously, and treatments such as finasteride (a 5α-reductase inhibitor) and minoxidil (an AR antagonist) have already been clinically approved [17,37]. 

## 4. Materials and Methods

### 4.1. Materials and Reagents

LNCaP cells (a human prostate cancer cell line) and HDP cells (a human hair follicle dermal papilla cell line) were bought from the American Type Culture Collection (Rockville, MD, USA). Testosterone, finasteride, sesame oil, and dimethyl sulfoxide (DMSO) were purchased from Sigma (St. Louis, MO, USA). RPMI 1640 medium, trypsin (0.25%), and penicillin-streptomycin solution were purchased from HyClone Laboratories (Logan, UT, USA). CEFOgro^TM^ Human Dermal Papilla Growth Medium (HDP growth medium) was obtained from CEFO Co. (Seoul, Korea). FBS was bought from Gibco (Grand Island, NY, USA). Phosphate-buffered saline was bought from Samchun Pure Chemical Co. (Gyeonggi-do, Korea). TRIzol reagent was bought from Molecular Research Center, Inc. (Cincinnati, OH, USA). Antibodies for Bcl-2, Bax, caspase 3, and cleaved caspase 3 were obtained from Cell Signaling Technology (Beverly, MA, USA), and antibodies for GAPDH and AR were bought from Santa Cruz Biotechnology, Inc. (Dallas, TX, USA).

### 4.2. Preparation of C. semidecandrus Ethanol Extract and GC-MS

*Connarus semidecandrus* was a kind gift from the National Institute for Biological Resources (Incheon, Korea). The whole *C. semidecandrus* plant was pulverized and then granulated for 24 h at 20–22 °C using 70% EtOH. For complete removal of EtOH, a rotary flash evaporator (Büchi Labortechnik AG, Flawil, Switzerland) was used for filtering and concentration under a vacuum at 10 h Pa and 40 °C. Further evaporation of the aqueous solution was conducted at 5 m Torr and −85 °C, and then the extract was lyophilized [38]. GC-MS was conducted in the Cooperative Center for Research Facilities of SKKU (Gyeoggido, Korea).

### 4.3. Animals and Cell Culture 

C57BL/6 mice (male, five weeks old) were purchased from Orient Bio (Iksan, Korea) and raised in plastic cages with plenty of water and food. Guidelines from the Institutional Animal Care and Use Committee at Sungkyunkwan University were followed in this study (SKKUIACUC-2021-07-18-1).

LNCaP cells were cultured in RPMI 1640 medium containing 10% FBS and 1% antibiotics (penicillin and streptomycin) at 37 °C, and HDP cells were cultured in HDP growth medium at 37 °C. Both cell lines were incubated with 5% CO_2_.

### 4.4. Testosterone-Induced AGA Model 

**Figure 7:**
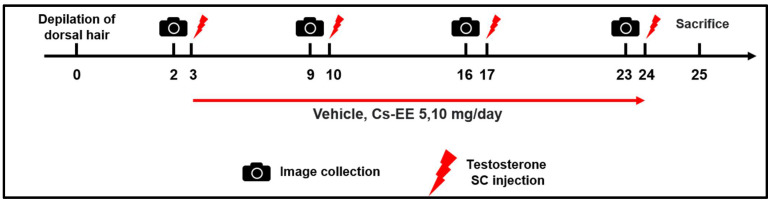
General scheme of the in vivo experiment. Shaved mice were given a subcutaneous injection of testosterone (40 μg/kg) four times over the course of three weeks, and Cs-EE (0–10 mg/kg) was applied to the skin on the backs of the mice once a day for three weeks.

C57BL/6 mice (*n* = 6) were divided into five groups: the Normal group; the Vehicle (PBS: DMSO = 1:1) control group; the 40 μg/kg/week testosterone + Cs-EE 5 mg/day group; the 40 μg/kg/week testosterone + Cs-EE 10 mg/day group; and the 40 μg/kg/week testosterone + finasteride 5 mg/kg/day group (positive control) (Figure 7). To evaluate the hair-growthpromoting effect of Cs-EE, the dorsal part of each mouse was treated with hair removal cream and shaved using an electric clipper twice, seven and three days before the first testosterone treatment. 40 μg/kg of testosterone dissolved in sesame oil was subcutaneously injected once a week four times. Cs-EE (5 and 10 mg/kg) or finasteride (5 mg/kg) diluted with PBS and DMSO (ratio of 1:1) were applied to the skin on the back every day during the three weeks of the testosterone injection period. One day after the last testosterone injection, all groups of C57BL/6 mice were euthanized; the dorsal hairs were isolated from the mice to confirm hair type frequency and hair thickness.

### 4.5. Hair–Skin Ratio, Hair Type Frequency, and Hair Thickness Analysis

After shaving the mouse hair, pictures of the dorsal skin were collected every week, and hair growth areas were scored with numbers from 1 to 10 depending on their color using ImageJ. Then, up to 350 hairs per mouse were collected to measure the frequency of hair type (guard, awl, auchene, and zigzag) [39], andthe thickness of each hair was measured using micrometer vernier calipers from ANENG (Guangdong, China).

### 4.6. Hematoxylin & Eosin Staining

Mouse back skins were fixed in 3.7% formalin for two days at 4 °C and then embedded in paraffin. After sectioning (4 μm), hematoxylin and eosin (H&E) were used to stain the tissue for hair-loss examination.

### 4.7. Ex Vivo Culture of C57BL/6 Mouse Hair Follicles

Five-week-old C57BL/6 mice were euthanized to obtain vibrissae hair follicles (*n* = 15) [40]. The hair follicles were cultured in HDP growth medium at 37 °C and 5% CO_2_. One day later, after media suction, either DMSO or Cs-EE (100 μg/mL) was administered to the hair follicles, and the hair length was measured at 0, 8, 16, 24, and 48 h after seeding.

### 4.8. Quantitative Real-Time PCR

RNA from mouse skin tissue from the alopecia model and total RNA prepared from HDP cells treated with DHT (100 nM) for 24 h after pre-treatment with 25, 50, or 100 μg/mL of Cs-EE were extracted using TRIzol reagent. A cDNA synthesis kit from Thermo Fisher Scientific was used to synthesize cDNA from total RNA, as previously described [41,42]. The mRNA levels of Bcl-2, Bax, and caspase 9 (primers listed in Table 2 and Table 3) were measured using real-time PCR with SYBR premix Ex Taq. The expression levels were calculated relative to GAPDH. 

### 4.9. Western Blotting

The skin tissues from C57BL/6 mice were stored at −70 °C after being ground in liquid nitrogen. For use in Western blotting, the tissues were lysed with lysis buffer (50 mM Tris-HCl, pH 7.4; 120 mM NaCl; 25 mM β-glycerol phosphate, pH 7.5; 20 mM NaF; 2% Nonidet P-40; and protease inhibitors) as previously described [43,44]. After subsequent sonication, the lysates were pelleted by centrifuge at 12,000× *g* for 3 min and 4 °C. The resulting supernatants were used in the Western blotting analysis. Protein samples were analyzed using 10–15% SDS-polyacrylamide gel electrophoresis. The levels of AR, Bcl-2, caspase 3, cleaved caspase 3, and β-actin were measured with corresponding antibodies.

### 4.10. 5α—Reductase Activity Assay

The whole liver of a 10-week-old male Sprague Dawley rat was extracted and lysed with lysis buffer (7.5 nM K_2_HPO_4_, 3.25 nM KH_2_PO_4_, 1 mM DTT, 32 mM sucrose, 0.2 mM PMSF, and additional protease inhibitors) to obtain 5α-reductase [45,46]. The reaction was initiated by adding NADPH (34 mM), testosterone (0.4 mM), and McIlvaine buffer (pH 5.0) to 4 μL of the enzyme extract that had been treated with serial concentrations of Cs-EE. [47]. After incubation, the reaction was stopped by heating for 5 min at 80 °C. The absorbance was measured at 340 nm to detect the oxidation of NADPH.

### 4.11. Cell Viability Assay

LNCaP and HDP cells were cultured in 96-well plates at concentrations of 1 × 10^4^ cells/mL or 5 × 10^4^ cells/mL cells/mL. Cs-EE (50 μL) was added to each well. After 24 h, 10 μL of 3-(4,5-dimethylthiazol-2-yl)-2,5-diphenyl tetrazolium bromide (MTT) solution was added, and the cells were cultured for 3h before 100 μL of 10% SDS in 0.01 M HCl was added to each well for 24h to stop the reaction and dissolve the formazan [48,49]. Subsequently, the absorbance of the MTT formazan was measured at 540 nm.

### 4.12. Cell Proliferation Assay

A proliferation assay was conducted in both the presence and absence of DHT. LNCaP and HDP cells were separately cultured at 3 × 10^3^ cells/mL in 96-well plates, and cell viability was measured at 0, 24, 48, and 72 h after Cs-EE treatment [50]. The optical density was detected at 540 nm.

### 4.13. Statistical Analysis

All experiments were at least duplicated independently for statistical comparisons of the mean ± standard deviation. For statistical comparison, the data were analyzed with *t*-testing. *P*-values of less than 0.05 were considered statistically significant.

## 5. Conclusions

**Figure 8:**
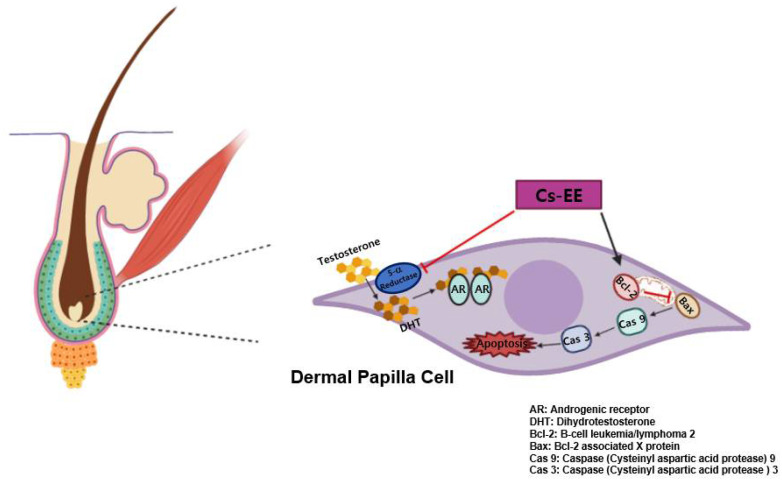
Schematic representation showing the anti-androgenic and anti-apoptotic effects of Cs-EE.

Numerous efforts to find anti-alopecia plant extracts have previously been conducted [51,52,53]. Here, we found that Cs-EE can alleviate 5α reductase activity, which inhibited the protein expression of AR. It was also demonstrated that Cs-EE wasable to reduce the intensity of the caspase cascade by increasing Bcl-2 at both the mRNA and protein levels in vitro, and enhanced hair growth and thickness in vivo (Figure 8). These results strongly suggest that Cs-EE has anti-alopecia potential and can be further clinically applied for men with hair loss as a topical medication such as ointment, gel, or cream for AGA treatment. In addition, because Cs-EE suppressed the proliferation of LNCaP cells, an androgen-sensitive human prostate cancer cell line, the anti-prostate cancer effect of Cs-EE should be continuously evaluated. Finally, the active ingredients of Cs-EE that show anti-alopecia activity will be identified in a subsequent project.

## Figures and Tables

**Table 1:** Phytochemical analysis of *C. semidecandrus* Jack ethanol extract by GC-MS.

Peak No.	Retention Time	Name of the Compound	Peak Area %
1	1.768	Acetic acid	7.31
2	2.084	2-Propanone, 1-hydroxy	2.74
3	3.250	Glyceraldehyde	1.30
4	3.588	2-Furanmethanol	1.45
5	3.848	1,4-Butanediamine	1.95
6	4.022	Dihydroxyacetone	4.30
7	5.358	Glycerin	17.05
8	6.576	Maltol	2.21
9	7.472	Ethanamine, N-ethyl-N-nitroso-	1.21
10	7.616	4H-Pyran-4-one, 2,3-dihydro-3,5-dihydroxy-6-methyl	5.09
11	7.802	Benzoic acid	2.74
12	8.356	Catechol	0.92
13	8.628	2-Pyrrolidinone	1.56
14	8.776	5-Hydroxymethylfurfural	3.94
15	9.007	1,2,3-Propanetriol, 1-acetate	2.64
16	9.533	4H-Pyran-4-one, 2,3-dihydro-3,5-dihydroxy-6-methyl	3.16
17	10.046	Phosphoramidothioic dichloride, dimethyl	0.86
18	10.425	2,7-Oxepanedione	3.42
19	10.526	Phenol, 2,6-dimethoxy	1.87
20	11.871	Ethanol, 2-(1-methylethoxy), acetate	14.01
21	13.685	4-Fluoro-N-(1H-tetrazol-5-yl)benzamide	4.53
22	14.042	Trehalose	6.97
23	14.246	2,6-Dimethoxyhydroquinone	4.59
24	15.219	4-((1E)-3-Hydorxy-1-propenyl)-2-methoxypehnol	0.52
25	17.409	*n*-Hexadecanoic acid	1.46
26	19.063	10(E),12(Z)-Conjugated linoleic acid	1.54
27	29.375	Tetrasiloxane, decamethyl	0.65

**Table 2:** Sequences of human primers used for PCR.

PCR Type	Gene Name	Sequence (5′-3′)	
Real-time PCR	Bcl-2 (Human)	Forward	ATCGCCCTGTGGATGACTGAGT
		Reverse	GCCAGGAGAAATCAAACAGAGGC
	Caspase 9 (Human)	Forward	AGGCAAGCAGCAAAGTTGTC
		Reverse	GTCTTTCTGCTCGACATCACCA
	GAPDH (Human)	Forward	GACAGTCAGCCGCATCTTCT
		Reverse	GCGCCAATACGACCAAATC

**Table 3:** Sequences of mouse primers used for PCR.

PCR Type	Gene Name	Sequence (5′-3′)	
Real-time PCR	Bcl-2 (Mouse)	Forward	GAGTACCTGAACCGGCATCT
		Reverse	GAAATCAAACAGAGGTCGCA
	Bax (Mouse)	Forward	GAACCATCATGGGCTGGACA
		Reverse	GGAGAGGAGGCCTTCCCAG
	Caspase 9 (Mouse)	Forward	CGAGAACTACCGCAGGAAGC
		Reverse	CTGTCGTATTCCCGCGATCC
	GAPDH (Mouse)	Forward	TGTGAACGGATTTGGCCGTA
		Reverse	ACTGTGCCGTTGAATTTGCC

## Data Availability

The data are contained within the article.

## Sample Availability

Samples of the compounds are not available from the authors.

## Abbreviations

Cs-EE	*Connarus semidecandrus* ethanol extract
AGA	Androgenic alopecia
AR	Androgenic receptor
DHT	Dihydrotestosterone
Bcl-2	B-cell leukemia/lymphoma 2
Bax	Bcl-2 associated X protein
Caspase 9	Cysteinyl aspartic acid protease 9
Caspase 3	Cysteinyl aspartic acid protease 3
LNCaP cell	Lymph node carcinoma of the prostate cell
HDP cell	Human hair follicle dermal papilla cell
GC-MS	Gas chromatography-mass spectrometry
MTT	3-(4,5-dimethylthiazol-2-yl)-2,5-diphenyl tetrazo
